# Influence of Fe Doping on the Electrochemical Performance of a ZnO-Nanostructure-Based Electrode for Supercapacitors

**DOI:** 10.3390/nano13152222

**Published:** 2023-07-31

**Authors:** Shalendra Kumar, Faheem Ahmed, Nagih M. Shaalan, Nishat Arshi, Saurabh Dalela, Keun Hwa Chae

**Affiliations:** 1 Department of Physics, College of Science, King Faisal University, P.O. Box 400, Al-Ahsa 31982, Saudi Arabia; fahmed@kfu.edu.sa (F.A.); nmohammed@kfu.edu.sa (N.M.S.); 2 Department of Physics, University of Petroleum & Energy Studies, Dehradun 248007, India; 3 Physics Department, Faculty of Science, Assiut University, Assiut 71516, Egypt; 4 Department of Basic Sciences, Preparatory Year Deanship, King Faisal University, P.O. Box 400, Al-Ahsa 31982, Saudi Arabia; nshastri@kfu.edu.sa; 5 Department of Pure & Applied Physics, University of Kota, Kota 324005, India; sdphysics@rediffmail.com; 6 Advanced Analysis & Data Center, Korea Institute of Science and Technology, Seoul 02792, Republic of Korea; khchae@kist.re.kr

**Keywords:** Fe-doped ZnO nanostructure, ferromagnetic, bound magnetic polaron, specific capacitance, electrochemical

## Abstract

ZnO is a potential candidate for providing an economic and environmentally friendly substitute for energy storage materials. Therefore, in this work, Fe-doped ZnO nanostructures prepared using the microwave irradiation procedure were investigated for structural, morphological, magnetic, electronic structural, specific surface area and electrochemical properties to be used as electrodes for supercapacitors. The X-ray diffraction, high-resolution transmission electron microscopy images, and selective-area electron diffraction pattern indicated that the nanocrystalline structures of Fe-doped ZnO were found to possess a hexagonal wurtzite structure. The effect of Fe doping in the ZnO matrix was observed on the lattice parameters, which were found to increase with the dopant concentration. Rods and a nanosheet-like morphology were observed via FESEM images. The ferromagnetic nature of samples is associated with the presence of bound magnetic polarons. The enhancement of saturation magnetization was observed due to Fe doping up to 3% in correspondence with the increase in the number of bound magnetic polarons with an Fe content of up to 3%. This behavior is observed as a result of the change in the oxidation state from +2 to +3, which was a consequence of Fe doping ranging from 3% to 5%. The electrode performance of Fe-doped ZnO nanostructures was studied using electrochemical measurements. The cyclic voltammetry (CV) results inferred that the specific capacitance increased with Fe doping and displayed a high specific capacitance of 286 F·g^−1^ at 10 mV/s for 3% Fe-doped ZnO nanostructures and decreased beyond that. Furthermore, the stability of the Zn_0.97_Fe_0.03_O electrode, which was examined by performing 2000 cycles, showed excellent cyclic stability (85.0% of value retained up to 2000 cycles) with the highest specific capacitance of 276.4 F·g^−1^, signifying its appropriateness as an electrode for energy storage applications.

## 1. Introduction

ZnO n-type semiconductors, due to their marvelous chemical stability, have largely been explored by the research community to discover their various functional utilizations in diverse fields, including spintronics, non-volatile magnetic data storage, etc. [1,2]. A large direct band gap and exciton binding energy (60 meV), along with optical transparency, makes ZnO advantageous for LED (light-emitting diode) and ETM (electron-transporting material) applications [3]. It is noteworthy that the electronic and optical response of ZnO can be elevated via doping with transition metals such as Fe, Mn, Co, Cr, etc. For instance, Ahmed et al. analyzed the effect of Co^2+^ inclusion on various properties of ZnO [4], and Kumar et al. studied the electronic structure of Fe-doped ZnO via X-ray spectroscopic analysis [5]. In this environment, TM- and rare-earth (RE)-doped metal oxides have been thoroughly researched. Substitution with transition metal ions may modulate the attributes of ZnO by creating variations in the structural parameters or inducing various vacancies, such as oxygen or Zn vacancies, without disturbing the parent stoichiometry. The partially filled d orbitals of transition elements are greatly influenced by their surroundings. ZnO doped with rare-earth ions is also of enormous interest for spintronic and optoelectronic applications since, as a result of intra-f-shell transitions, rare-earth ions have several unusual characteristics. Additionally, controlling the shape and size of doped ZnO nanocrystals shows great promise in possible applications such as nanoelectronics, sensors, and solar cells.

Moreover, ZnO, being a low-cost material with a thermodynamically stable structure and good redox capability, is one of the most promising electrode materials for supercapacitor applications. Continuous efforts are being undertaken to boost their energy density so that they can eventually replace batteries. Apart from this, ZnO is being further explored for electrochemical properties that can be utilized for supercapacitor applications [6,7,8,9,10,11,12]. It is well known that energy storage and transformation are emerging areas of concern for domestic as well as commercial usage. Electrical energy is conventionally stored in batteries, which have a higher energy density but exhibit a lower power density which is a drawback [13]. This problem can be solved using supercapacitors, which have proved to be ideal components of energy storage units. Supercapacitors are advantageous on account of their modifiable storage capacity and cyclic stability. The performance of supercapacitors can be enhanced by enhancing the specific capacitance, charging–discharging rate, and cyclic stability of the material used. This helps improve the power density along with the energy density.

Based on their function, supercapacitors may be classified as double-layered or pseudocapacitors. The operation of double-layer supercapacitors is based on the formation of a double layer between the electrode and electrolyte, while the pseudocapacitors operate via Faradaic redox reactions. However, the operation of both of these types of supercapacitors is based on electrochemical performance, which means the storage of energy takes place via electrochemical reactions. The electrochemical reactions involve the redox cycle or charging–discharging cycle of the material [14]. Interestingly, ZnO is inexpensive, environmentally friendly, and highly chemically stable, having good electrochemical activity and display a high energy density of ~650 A·g^−1^. Despite these advantages, there is the drawback of a low cyclic life, which is associated with dendrite growth during the cycling process. The performance of ZnO can be improved, along with its electrochemical properties, by employing different methods and techniques for synthesis. Therefore, ZnO, as an electrode material, is widely used in supercapacitors. When considering the performance of ZnO as an electrode material in supercapacitor devices, intrinsic point defects play a critical role in both bulk and nanoscale ZnO. Selvakumar et al. found a specific capacitance of 160 F·g^−1^ for a ZnO-activated carbon (AC) composite with cyclic stability of 500 cycles prepared via the co-precipitation method [15]. Similarly, Lee et al. also investigated ZnO for its electrochemical performance, measuring a specific capacitance of 155 F·g^−1^ at 0.5 A·g^−1^ and a cyclic stability up to 1000 cycles [16]. In another study, the areal capacitance was measured to be 448 mF/cm^2^ with an energy density of 0.12 mW h/cm^3^ [17]. For enhancing the performance of supercapacitors, transition metal elements may be doped in ZnO, which can further enhance the chemical reactivity of the material and, hence, the redox properties.

Therefore, in this work, we have synthesized Fe-doped ZnO nanostructure using the microwave irradiation technique with varying concentrations of Fe to investigate the effect of Fe concentration on its structural, morphological, electronic structural, magnetic, and electrochemical properties. From the viewpoint of the electronic structural properties of these nanostructures, no agreement has been reached on the genesis of ferromagnetic properties. Hence, in this manuscript, we undertake a fundamental, systematic, and detailed study on the electronic structure and magnetic properties of Fe-doped ZnO nanostructures. Further, this study is meant to enhance the understanding of electrochemical properties for the improved performance of supercapacitors as an electrode material at the nanoscale. We expect that the outcomes of our study will provide some plausible and conclusive evidence for new developments in the pursuit of research advancements in this field.

## 2. Experimental Section

**Chemicals Required:** All chemicals, including zinc acetate, iron acetate, and potassium hydroxide, were procured from CDH. The chemicals used were of a 99.9% purity and employed as acquired, without any further purification.

**Scheme 1:**
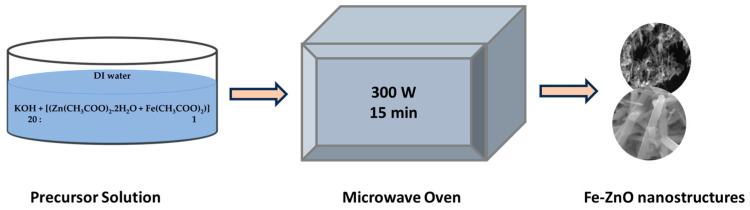
The schematic shows the synthesis procedure followed for the preparation of the nanoparticles.

**Synthesis Procedure:** The microwave irradiation process was utilized for preparing the Fe-doped ZnO nanostructures, as shown in Scheme 1. All samples were prepared by using a domestic microwave oven (Samsung, 750 W). In the typical synthesis of Fe-doped ZnO nanostructures, the molar ratio of KOH to (Zn(CH_3_COO)_2_·2H_2_O + Fe(CH_3_COO)_2_) was maintained at 20:1, and then the mixture was prepared in 100 mL of distilled water. Finally, the mixture was kept for irradiation in a microwave, operated at 300 W for 15 min, and thereafter, the solution was allowed to cool until it reached room temperature. The precipitate obtained after microwave irradiation was washed using deionized water and absolute ethanol, and finally, was kept for drying in a hot air oven at 80 °C for 12 h.

**Characterization Techniques:** A Bruker-D8 X-ray diffractometer with Cu Kα radiation (λ = 1.5406 Å) was used for crystal structure analysis and determining phase purity. The field-emission scanning electron microscopy (FESEM) measurements were carried out using an MIRA II LMH microscope. The elemental composition analysis was performed using energy-dispersive X-ray spectroscopy (EDX, Inca Oxford). A microscope of JEOL (JEM-2100F version operated at 200 kV) was employed for capturing transmission electron microscopy images and high-resolution transmission electron microscopy (HR-TEM) images, along with identifying the selected-area electron diffraction (SAED) pattern. The surface area of the Zn_0.97_Fe_0.03_O nanostructures was determined using the Brunauer–Emmett–Teller (BET) method. The data were collected by purging N_2_ gas at a temperature of up to 1000 °C in the air at a heating rate of 10 °C/min using a surface area analyzer (ASAP 2020, Micromeritics). Magnetization properties at room temperature were studied using a commercial Quantum Design physical property measurement system. The 10D XAS KIST (Korea Institute of Science and Technology, Seoul, Republic of Korea) soft X-ray beamline of Pohang Accelerator Laboratory (PAL) was used to study Fe L_3.2_ and O K-edge spectra.

Electrochemical measurements, such as cyclic voltammetry (CV), galvanostatic charge–discharge (GCD), and electrochemical impedance spectroscopy (EIS), of Fe-doped ZnO nanostructures were performed in three electrode configurations using the Corrtest-CS150 electrochemical analyzer at room temperature. The working electrodes were designed using Fe-doped ZnO nanostructures, polyvinylidene fluoride (PVDF), and carbon black in the ratio of 80:10:10, respectively, along with NMP. The nickel foam was homogeneously coated with the mixture, and the electrode was dried in a hot air oven at 90 °C for 24 h. All of the electrochemical measurements were performed with a 1 M KOH electrolyte with a Pt wire acting as a counter electrode and Ag/AgCl serving as the reference electrode in a potential window from 0 to 0.65 V. The specific capacitance (C_S_) of the Fe-doped ZnO nanostructures was determined using the equation [13]:

(1) $$C_{s}=\frac{1}{m\nu \Delta V}\int \;IdV,$$

where $C_{s}$ is the specific capacitance, I shows the charging current, ΔV highlights the voltage range, and ν and m denote the scan rate and the mass of the active electrode, respectively. Furthermore, a galvanostatic discharge plot was used to calculate the specific capacitance of the Fe-doped ZnO nanostructures using the equation:

(2) $$C_{s}=\frac{I\cdot \Delta t}{\Delta V\cdot m},$$

where Δt signifies the discharge time, I is the current density, m denotes the active material deposited on the working electrode, and ΔV symbolizes the range of the potential used in the charge–discharge measurements.

## 3. Results and Discussion

**Figure 1:**
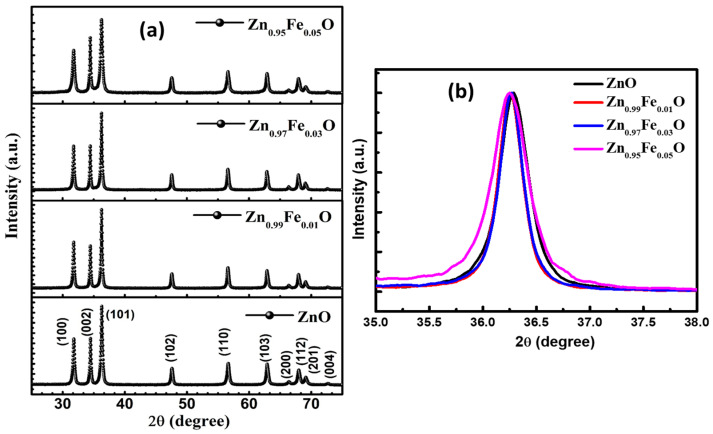
(**a**) X-ray diffraction patterns of undoped and Fe-doped ZnO nanostructures; (**b**) enlarged view of high-intensity peaks shifting towards lower 2θ values.

X-ray diffraction (XRD) patterns of Fe-doped ZnO nanostructures measured in θ–2θ mode are displayed in Figure 1a. It can be noticed that all of the observed reflections correspond to the wurtzite structure, and the absence of any alien peak rules out the possibility of secondary phases. The peak positions were found to shift towards the lower 2θ value with an increasing Fe dopant amount in the ZnO nanostructures (see Figure 1b), which infers that the lattice parameters increase with doping. The measures of the crystallographic parameters were evaluated using the Powder-X software. The lattice parameter values, i.e., a and c, were estimated to increase from 3.249 Å to 3.252 Å and 5.202 Å to 5.208 Å, respectively, with an uncertainty of ±0.0002. The results observed here are analogous to the earlier reports. It is worth saying that the shifting of peaks towards the lower 2θ value due to Fe doping signifies an increase in the lattice constant with doping. The volumes of unit cells calculated using the lattice parameters were found to be 47.554 Å^3^, 47.602 Å^3^, 47.669 Å^3^, and 47.756 Å^3^ for pure ZnO and for 1%, 3%, and 5% Fe-doped ZnO, respectively. The shift in XRD peaks and the increasing behavior of the lattice parameters *a* and *c*, allow us to infer that the doped Fe ions were effectively substituted in place of the Zn ions in the parent ZnO lattice, retaining its hexagonal wurtzite structure.

**Figure 2:**
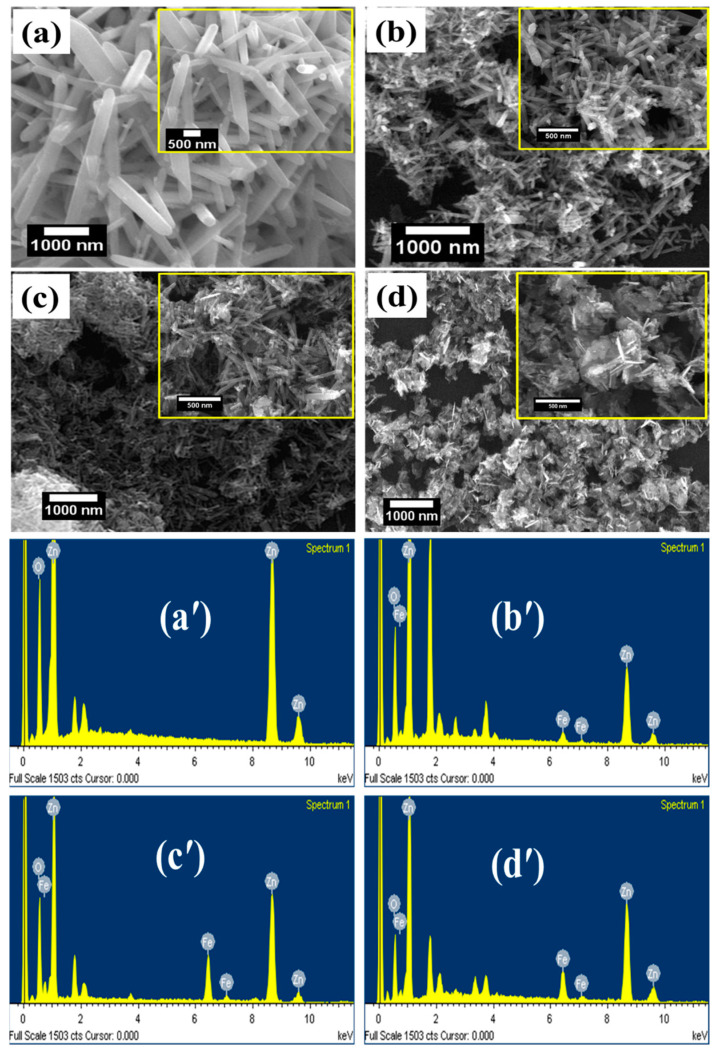
Field-emission scanning electron microscopic images of (**a**) ZnO, (**b**) Zn_0.99_Fe_0.01_O, (**c**) Zn_0.97_Fe_0.03_O, and (**d**) Zn_0.95_Fe_0.05_O; (**a′**–**d′**) electron-dispersive X-ray spectra of undoped and Fe-doped ZnO nanostructures.

The surface morphology of the Fe-doped ZnO nanostructures was analyzed using field-emission scanning electron microscopy (FESEM) measurements, as highlighted in Figure 2a–d. It can be seen that undoped ZnO displays a nanorod-type morphology. The dimensions of the ZnO nanorods, such as diameter and average length, were observed to be ~200–250 nm and ~1.5 μm, respectively. Furthermore, Figure 2b–d presents the FESEM images of 1%, 3%, and 5% Fe-doped ZnO nanorods, respectively. It is evident from Figure 2b,c that 1% and 3% Fe—doped nanostructures additionally display rod-like morphology having diameter and average length of ~75 nm and ~450 nm, respectively. It is noticeable from Figure 2d that 5% Fe-doped ZnO nanostructure exhibits a nanosheet-type morphology with a thickness of ~35 nm and a lateral dimension of ~350 nm. It is worth mentioning here that the sizes of the nanostructures decrease with Fe doping, which indicates that Fe ions hinder growth. Additionally, the energy-dispersive X-ray (EDX) was utilized for compositionally analyzing the Fe-ZnO nanostructures. Figure 2a′–d′ displays the EDX spectra of Fe-doped ZnO nanostructures. It is evident from the EDX spectra that the pure ZnO nanostructure demonstrates the presence of Zn and O peaks only, whereas the Fe-doped nanostructures display the peaks corresponding to Fe along with Zn and O.

**Figure 3:**
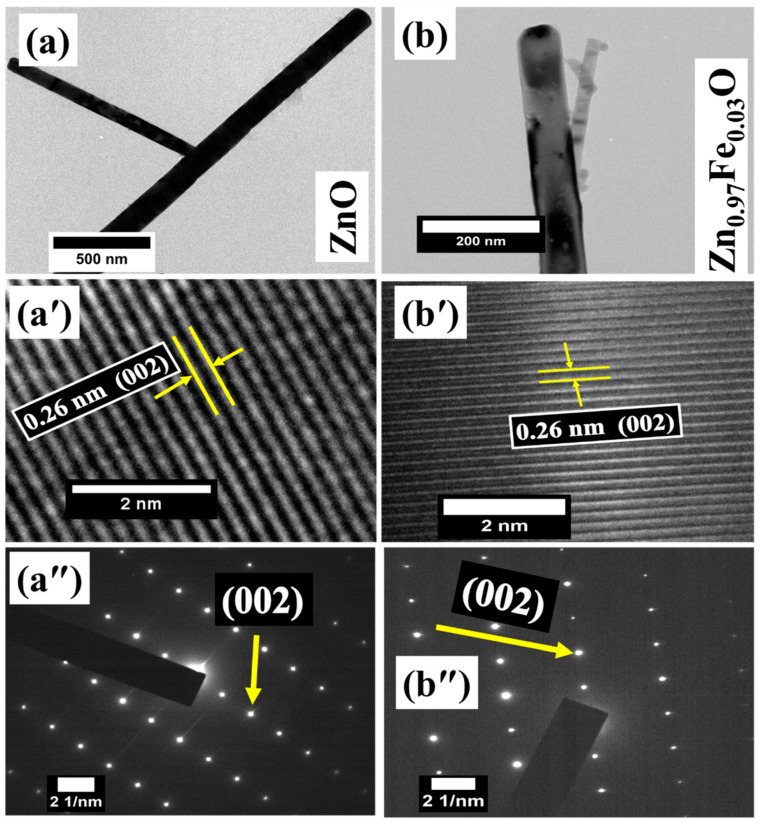
(**a**,**b**) TEM micrographs of ZnO and Zn_0.97_Fe_0.03_O nanostructures, (**a′**,**b′**) HR-TEM images of ZnO and Zn_0.97_Fe_0.03_O nanostructures, and (**a″**,**b″**) the SAED pattern of ZnO and Zn_0.97_Fe_0.03_O nanostructures.

The transmission electron microscope (TEM) was further unitized for the surface morphology analysis. Figure 3a,b shows the morphology of the ZnO and Zn_0.97_Fe_0.03_O nanostructures. It is evident from the TEM micrographs that the pure ZnO and Fe-doped ZnO samples display a rod-type morphology, which is in good agreement with the results obtained from the FESEM micrographs. Furthermore, the phase purity of the ZnO and Zn_0.97_Fe_0.03_O nanostructures was determined using HR-TEM micrographs and the SAED pattern, as displayed in Figure 3a′,b′,a″,b″. The interplanar spacing of the undoped ZnO and Fe-doped-ZnO nanostructures was measured with the help of the HR-TEM pattern (see Figure 3a′,b′) and was observed to be ~0.26 nm. The value of the interplanar distance matches well with the ZnO (002) plane of the hexagonal wurtzite crystal structure. In addition, the SAED pattern also indicates the single-phase nature of the ZnO and Zn_0.97_Fe_0.03_O nanostructures and agrees well with the XRD results, rejecting the presence of any impurity phase.

**Figure 4:**
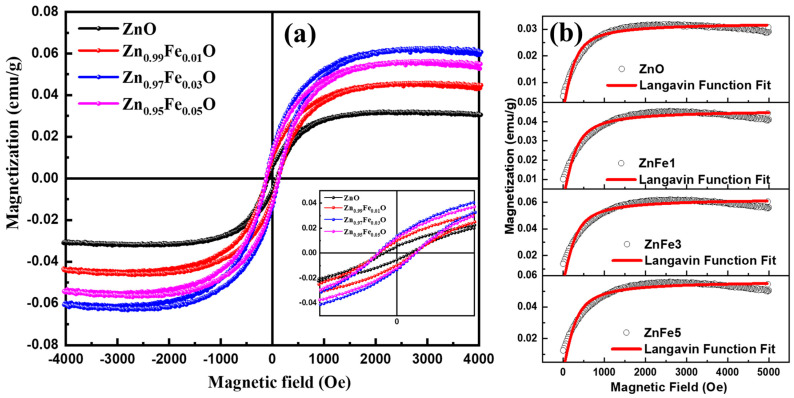
(**a**) M-H hysteresis curves with inset showing the near H = 0 behavior and (**b**) fitting of high-field M-H curves with Langevin function of undoped and Fe-doped ZnO nanostructures.

The room-temperature magnetization measurements of the Fe-doped ZnO nanostructures, as presented in Figure 4a, were carried out to understand their magnetic behaviors. It can be seen that the compositions demonstrate weak ferromagnetic ordering at room temperature. It should be noted that the coercive field (H_C_) and remanent magnetization (M_r_) of Fe-doped ZnO nanostructures were found in the range of 83 Oe to 124 Oe and 5.3 × 10^−3^ to 12.5 × 10^−3^ emu/g, respectively. Furthermore, the values of the saturation magnetization (M_S_), determined with the M-H loop, were observed in the range of 3.1 × 10^−2^ to 5.3 × 10^−2^ emu/g. It is worth mentioning that various magnetic properties increased until 3% Fe doping was reached, and thereafter, decreased with a further increase in the doping. There are various reports that have claimed that there is intrinsic ferromagnetism in TM-doped ZnO [18,19,20], whereas some other reports have demonstrated that ferromagnetism is caused by a secondary phase or defects [21]. Liu et al. [22] studied an Fe-doped ZnO nanocrystal and suggested that the observed room-temperature ferromagnetism is an intrinsic property of the material. Furthermore, some reports have demonstrated that the doping of transition metals, such as Mn and Fe, does not play any role in the magnetic properties of doped ZnO. Additionally, in one study, the ferromagnetic ordering in a chemically synthesized Fe-doped ZnO nanostructure was described based on the zinc vacancy (Z_ni_) [23], whereas ferromagnetism in Fe-doped ZnO thin films was attributed to the secondary phase of a high doping concentration. Furthermore, the M-H hysteresis curves were fitted using the BMP model (see Figure 4b) to understand the room-temperature magnetization in Fe-doped ZnO using Equations (3)–(5) [24,25]:

M = M_o_ L(x) + χ_m_ H; (3)

L(x) = Mo [coth(x) − 1/x];(4)

(5) $$x=\frac{m_{\mathrm{eff}}H}{k_{B}T}$$

In the above expression, Equation (3), the first term is the BMP contribution, while the second term represents the paramagnetic contribution. M_o_ is the multiplication of the effective spontaneous moment (m_eff_) per BMP and the number of BMPs per unit volume (N). The fitting with the above expression for both types of contributions did not converge. Then, the second term was removed, and the curves were fitted with the term considering only the BMP contribution. This time, the fitting was converged, and the values of the parameters M_o_ and m_eff_/k_B_T were obtained, as represented in Table 1. Using these parameters, the values of N and m_eff_ were obtained and are represented in Table 1. It can be seen that the values of M_o_, N, and m_eff_ increase with an Fe doping up to 3%, beyond which they either decrease or remain consistent. The increase in magnetization, the effective magnetic moment, and the number of BMPs per unit volume may be associated with the increase in the concentration of Fe ions. This infers that the increase in Fe concentration is related to the formation of BMPs, which is responsible for the ferromagnetic response. The values of m_eff_ (~10^−16^ emu) for Fe-doped ZnO are larger, while the number of BMPs per unit volume (~10^14^ cm^−3^) in Fe-doped ZnO is smaller as compared to the reported values of Ni-doped ZnO [24]. Maibam et al. [26] also explained the ferromagnetism in Fe-doped ZnO nanostructures synthesized using a hydrothermal method based on the BMP model. Particle size, annealing temperature, intrinsic properties of the doped ions, secondary phases, defects and impurities, positions of the dopant ions, distances between dopant ions, etc., are the primary factors that determine the ferromagnetic ordering. The ferromagnetic characteristics of the materials are generated by the mutual interaction of BMPs, which align the spins of charge carriers along the field direction. As a result, larger amounts occupied by BMPs have a tendency to overlap with the BMPs, leading to weak ferromagnetic behavior in these samples.

**Figure 5:**
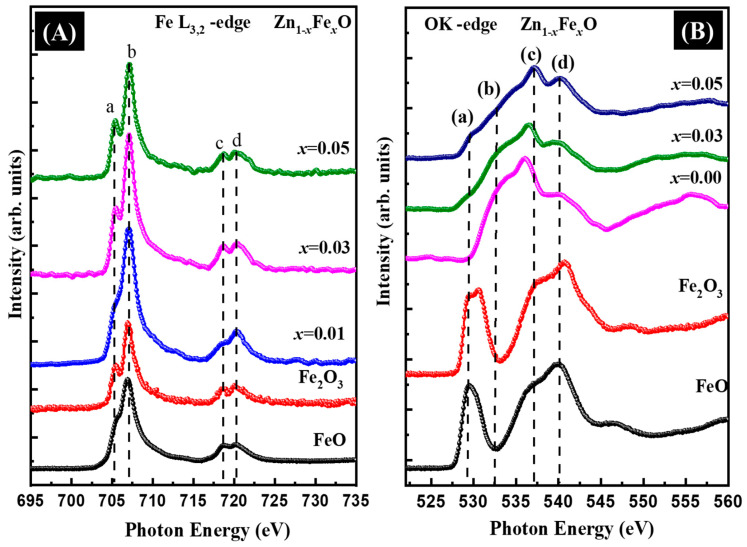
Near-edge X-ray fine spectra obtained at (**A**) L_3,2_ edge, and (**B**) O K edge of undoped and Fe-doped ZnO nanostructures.

Near-edge X-ray absorption fine-structure (NEXAFS) spectroscopy was employed for studying the electronic structure of Fe-doped ZnO nanostructures. The NEXAFS is an excellent technique for determining the valance state of the probed ion in the samples. The NEXAFS spectra of Fe-doped ZnO nanostructures at the Fe L_3,2_ edge have been recorded along with the reference spectra of FeO and Fe_2_O_3_ samples. Figure 5A demonstrates the Fe L_3,2_-edge spectra of Fe-doped ZnO nanostructures. The shape and intensity of the spectral lines provide information about the coordination of the Fe ions in the host matrix. The intensity of the Fe L_3,2_-edge spectral features indicates the number of total unoccupied Fe 3d states. Two sets of peaks are visible in the spectra and are appointed as Fe 2p_3/2_ (a,b) and Fe 2p_1/2_ (c,d), which correspond to the L_3_ and L_2_ edges, respectively. Each of these edges further split into two (t_2g_ and e_g_), which is attributable to ligand-field splitting. The spectral features of the Fe L_3,2_-edge NEXAFS spectra mainly originate due to the hybridization of Fe 2p-3d orbitals, which are highly affected by the core-hole potential. These peaks arise from excitations that move from Fe 2p-filled core states to unoccupied Fe 3d states, which is why the NEXAFS spectra provide a measure of unoccupied states. The two well-known L_3_ and L_2_ multiple-structure spectra can be clearly seen in the reference spectra of the FeO and Fe_2_O_3_ compounds. A difference in the L_3_ features of the FeO (Fe^2+^) and Fe_2_O_3_ (Fe^3+^) compounds is observed, which is due to the variation in the valence states of Fe ions. Well-developed doublet-like features in the L_3_ edge of Fe_2_O_3_ are observed, which can be assigned to the Fe t_2g_ and e_g_ orbitals, respectively. The shoulder towards the right of edge L_3_ (peak a) is observed as becoming more intense as the amount of Fe increases in the sample. The change in the intensity of the shoulder peak indicates the change in the oxidation state of Fe with respect to the increase in the amount of Fe in the host lattice. Moreover, it can be seen that in the case of FeO reference samples, the first peak turns into the shoulder of the main peak and matches the spectra of Fe-doped ZnO that has a 1% amount of Fe. This means that the Fe is present in the +2 oxidation state in the 1% Fe-doped ZnO sample. It can be noticed that the spectral features observed at the Fe L_3,2_ edge of the Fe-doped ZnO nanostructures match well with Fe_2_O_3_ as the amount of Fe increases to 5%, suggesting that most of the Fe ions in the ZnO matrix are in the +3 valence state. Furthermore, the main peak of the L_3_ edge (peak b) provides information about the local electronic environment of the probed ion. The results obtained in the present work are analogous to those from earlier reports. Gautam et al. [27] carried out an electronic structure study of Fe-doped ZnO nanorods using X-ray absorption fine-structure (EXAFS) spectroscopy, in which they observed that Fe (Fe^3+^/Fe^2+^) ions are in mixed valence states. Kumar et al. [5] discussed their NEXAFS study carried out at the Fe L_3,2_ edge, and they demonstrated that the majority of the Fe ions are in the Fe^+3^ valence state in Fe-doped ZnO nanorods. Similarly, Fe-doped ZnO nanocrystals were studied by Jang et al. [28], who stated that the majority of Fe ions are in the Fe^+3^ valence state.

Figure 5B shows the O K-edge spectra of Fe-doped ZnO recorded in total electron yield (TEY) mode, along with Fe_2_O_3_ and FeO as reference compounds. The spectral features recorded in the O K-edge spectra originate because of an O 1s to O 2p transition, i.e., O 1s filled states make transition into O 2p unoccupied states. The dipole-allowed spectral characteristic of metal oxides observed in O K-edge spectra suggests unoccupied states in the conduction band and its hybridization with diverse Zn and Fe orbitals. The spectral characteristic that originates in the range of 530–535 eV is due to a transition from the O 1s to O 2p orbital that is hybridized with Fe 3*d* orbitals. Peaks a and b may be assigned the O 2p weight in the Fe *3d* band. The fainted intensity of these peaks may lead to completely occupied higher orbitals. The spectral feature observed in the higher energy region, i.e., after 545 eV, originates because of a transition from the 1*s* core state to the O 2*p* states that are hybridized with extended Fe 4*sp* orbitals. It should be noted that the intensity of the spectral features increases with doping, which clearly signifies that Fe ions are successfully replaced by Zn ions in the ZnO host matrix.

**Figure 6:**
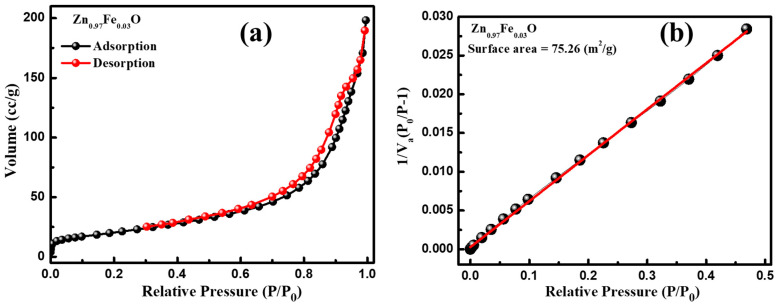
(**a**,**b**) N_2_ adsorption–desorption BET isotherms of Zn_0.97_Fe_0.03_O nanostructures.

The BET method was employed for the specific surface area calculation of the Zn_0.97_Fe_0.03_O nanostructures. Figure 6a represents the N_2_ adsorption–desorption isotherm curve of the Zn_0.97_Fe_0.03_O nanostructures. The surface area of the Zn_0.97_Fe_0.03_O nanostructures was observed to be 75.26 m^2^/g (see Figure 6b), which is higher than that of the pure ZnO nanostructures reported in the literature [29,30]. Ahmad et al. [29] described the surface area of yttrium- and cerium-doped ZnO nanoparticles, which they determined using the BET method. They observed a specific surface area of 63.32 m^2^/g for pure ZnO nanoparticles. In another report, Hui et al. [30] prepared Fe-doped ZnO nanoparticles and found that the specific surface area increases from 13.4 m^2^/g to 24.8 m^2^/g for 5% Fe-doped ZnO nanoparticles. In this work, the higher value of the specific surface area of 75.26 m^2^/g in the Fe-doped ZnO nanostructures might be due to the formation of defects (oxygen vacancies). It is worth mentioning here the improved electrochemical performance of Zn_0.97_Fe_0.03_O nanostructures, such as specific capacitance and cyclic stability. might be due to its higher specific surface area value. The larger specific surface area may increase the active sites and deliver good contact between the electrolyte and electrode material.

**Figure 7:**
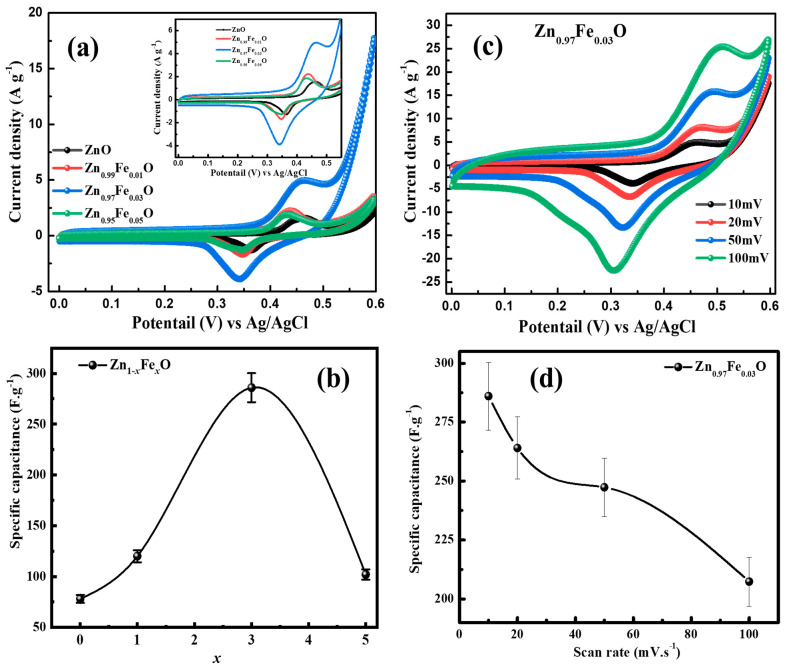
(**a**) CV plots of Fe-doped ZnO nanostructures at scan rate of 10 mV·s^−1^, (**b**) variation in specific capacitance of Fe-doped ZnO nanostructures at scan rate of 10 mV·s^−1^, (**c**) CV plots of Zn_0.97_Fe_0.03_O nanostructures with different scan rates, (**d**) variation in specific capacitance of Fe-doped ZnO nanostructure electrodes with different scan rates.

We carried out electrochemical measurements to determine the electrode performance of Fe-doped ZnO nanostructures for energy storage applications. Figure 7a demonstrates the cyclic voltammetry (CV) curve of Fe-doped ZnO nanostructures captured at a scan rate of 10 m·Vs^−1^. The potential window from 0.0 to 0.60 V was utilized for the recording of all of the CV curves. The current response of Fe-doped ZnO electrodes measured at 10 m·Vs^−1^ was observed to increase with the Fe concentration, which clearly indicates the capacitive behavior of all of the electrodes. The peaks obtained from the CV curves during charging and discharging cycles can be associated with the reactions shown in Equations (6) and (7) [31]:

(6) $$\mathrm{ZnO}+H_{2}O+2e^{-}\;\overset{Charging/Discharging}{↔}\;{\mathrm{Zn}}_{2}O+2{\mathrm{OH}}^{-}$$

(7) $$\mathrm{FeO}+H_{2}O+2e^{-}\;\overset{Charging/Discharging}{↔}\;\mathrm{Fe}\;(\mathrm{OH}){}_{2}+{\mathrm{OH}}^{-}$$

**Figure 8:**
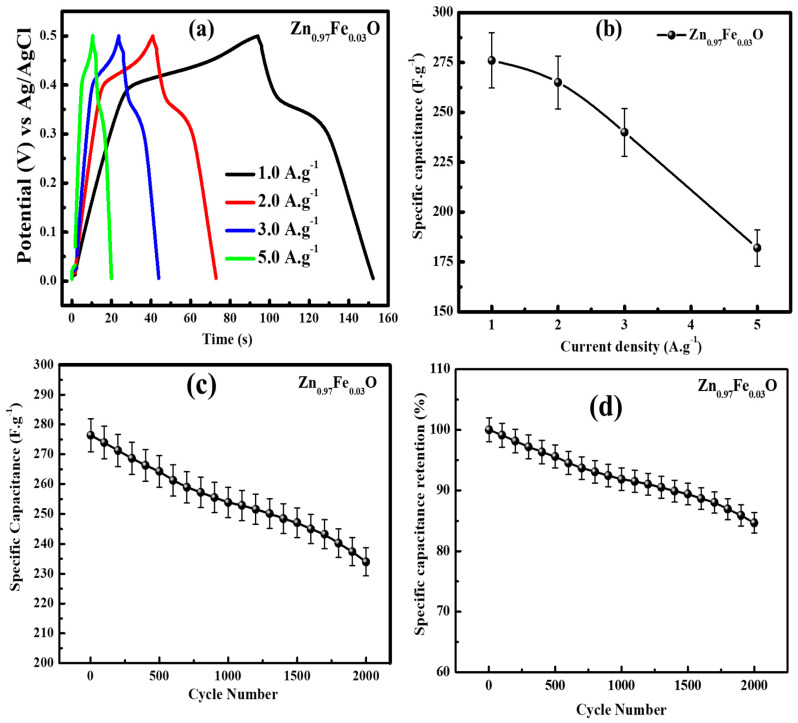
(**a**) Galvanostatic charge–discharge plots of Zn_0.97_Fe_0.03_O nanostructures recorded at different current densities, (**b**) variation in specific capacitance of Zn_0.97_Fe_0.03_O nanostructures at different current densities, (**c**) cycle stability of Zn_0.97_Fe_0.03_O electrodes at a current density of 1 A·g^−1^, and (**d**) specific capacitance retention (%) of Zn_0.97_Fe_0.03_O electrodes at a current density of 1 A·g^−1^.

Furthermore, it is worth mentioning here that the area under the CV curve increased with Fe doping and displayed the maximum area under the curve for the 3% Fe-doped ZnO electrode, and then it started decreasing as the Fe concentration increased further. The specific capacitance (C_s_) of Fe-doped ZnO electrodes calculated using Equation (1) with the help of the CV plot is depicted in Figure 7b. The Cs values were found to be 78 F·g^−1^, 120 F·g^−1^, 286 F·g^−1^, and 102 F·g^−1^ for undoped and 1%, 3%, and 5% Fe-doped ZnO electrodes, respectively. Additionally, the CV measurements of the 3% Fe-doped ZnO electrode were measured at various scan rates of 10, 20, 50, and 100 m·Vs^−1^ (see Figure 7c). It should be noted that the current response as well as the area under the CV curve were found to increase with the increase in the scan rate from 10 m·Vs^−1^ to 100 m·Vs^−1^. The Cs values of the 3% Fe-doped ZnO electrode that were calculated at different scan rates using Equation (1) were observed to decrease with an increasing scan rate. The values of Cs, as shown in Figure 7d, were found to be 286 F·g^−1^, 264 F·g^−1^, 247 F·g^−1^, and 207 F·g^−1^ at 10 m·Vs^−1^, 20 m·Vs^−1^, 50 m·Vs^−1^, and 100 m·Vs^−1^ scan rates, respectively, which were analogous to the reported results [32,33]. The lower value of Cs at a high scan rate is because the whole area of the active electrode is not attainable during the charge storage process due to inadequate amount of time [34]. The charging and discharging features of the 3% Fe-doped ZnO electrode were studied using the galvanostatic charge–discharge (GCD) plots. The GCD curves of the 3% Fe-doped ZnO electrode recorded in the potential window of 0–0.55 V at different current densities are displayed in Figure 8a. The values of Cs (see Figure 8b) were further determined from the GCD curve using Equation (2) at different current densities. Thus, the Cs value observed for the 3% Fe-doped ZnO electrode was ~276 F·g^−1^, which is analogous to the values determined using the CV plot. Various groups have studied and prepared ZnO-based electrodes and their electrochemical properties for supercapacitor applications. Hybrid MnOx-coated ZnO nanorod arrays were fabricated by Chen et al., and they observed a 222 F·g^−1^ specific capacitance at 25 mV·s^−1^ [35,36]. In another work. Wang et al. [36] opted for taking a green and facile approach for the preparation of graphene –ZnO nanocomposites and reported a specific capacitance of 62.2 F·g^−1^ at 0.5 A·g^−1^. A self-propagating solution combustion method was utilized by Jayalakshmi et al. for the synthesis of a zinc oxide/carbon (ZnO/C) composite, and then they studied its electrochemical properties. They observed a specific capacitance of 21.7 F·g^−1^ at a 50 mV·s^−1^ scan rate [37]. Furthermore, Lu et al. [38] prepared a graphene and graphene/ZnO nanocomposite using the microwave irradiation technique. They reported an improvement in the electrochemical performance of graphene/ZnO nanocomposites along with a specific capacitance of 146 F·g^−1^. In another report, ZnO-containing porous activated-carbon nanofibers were synthesized by Kim et al., and they found the specific capacitance value to be 178.2 F·g^−1^. Likewise, He et al. [39] prepared ZnO nanostructures with varying morphologies and then performed the electrochemical measurement. They reported that ZnO nanocones and ZnO nanowires displayed a specific capacitance value of 378.5 F·g^−1^ and 191.5 F·g^−1^, respectively. Additionally, Luo et al. [32] prepared an electrode using ZnO tetrapods and observed a specific capacitance of 160.4 F·g^−1^. Sahu et al. [13] synthesized Nd-doped ZnO nanoparticles using the microwave-assisted co-precipitation technique, and they observed a higher value of the specific capacitance of 154 F·g^−1^ for 3% Nd-doped ZnO nanoparticles. In this study, the cycle stability of a 3% Fe-doped ZnO electrode was determined by measuring 2000 cycles at a current density of 1.0 A·g^−1^, as highlighted in Figure 8c. It was found that the value of the specific capacitance is reduced from 276.40 F·g^−1^ to 134.0 F·g^−1^ after 2000 cycles. Figure 8d demonstrates the retention in the specific capacitance along with the GCD cycles and infers a 16% loss in capacitance. The values of the specific capacitance are displayed in Table 2 along with the values obtained from other compounds for the sake of comparison [40,41,42,43,44,45,46,47,48]. The electrochemical properties of the Fe-doped ZnO nanostructures show that Fe doping enhanced the electrode performance of ZnO. Therefore, by comparing our work with the previous reports mentioned, it can be seen that the 3% Fe-doped ZnO nanostructure electrode revealed outstanding cycle stability and that the electrode is suitable for supercapacitor application.

## 4. Conclusions

In summary, the microwave irradiation method was used to prepare undoped and Fe-doped ZnO nanostructures. The structural, morphological, magnetic, electronic structural, and electrochemical properties were investigated through various characterization techniques. The XRD, SAED and HR-TEM, confirm the hexagonal wurtzite structure of ZnO. Fe was found to be well substituted in the ZnO matrix, with a slight increase in the lattice parameters as well as in the cell volume. Field-emission scanning electron microscopy revealed the rod-like and nanosheet-like morphology of the nanostructures. Magnetization measurements revealed that the ferromagnetic nature originated due to the contribution of bound magnetic polarons. Saturation magnetization along with the number of bound magnetic polarons was found to increase with the addition of 3% Fe content, which was followed by a decrease that was attributable to the change in the oxidation state of Fe. The change in oxidation state was confirmed through the NEXAFS spectra, which showed that the Fe-doped ZnO matrix was in a mixed valence state (Fe^+2^/Fe^+3^) and the concentration of Fe^3+^ ions in ZnO increased with Fe doping. The surface area that was estimated using BET curves for the 3% Fe-doped ZnO nanostructures was found to be 75.25 m^2^/g. The Cs values determined using CV plots indicated that the Cs increased with Fe doping and showed a maximum value of 286 F·g^−1^ at 10 mV·s^−1^ for the 3% Fe-doped ZnO nanostructures. The cyclic measurements of the Zn_0.97_Fe_0.03_O electrode were performed for 2000 cycles at a current density of 1.0 A·g^−1^, which demonstrated noteworthy cyclic stability with an 85 % C_S_ retention up to 2000 cycles, indicating that this is an electrode that can be used in supercapacitor applications. In view of these results, Fe-doped ZnO nanostructures may be potential candidates for storage applications.

## Figures and Tables

**Table 1:** Parameters obtained from fitting of M-H curves in high-field range with Langevin function to find BMP contribution.

	Mo (emu/g)	m_eff_/k_B_T	N (cm^−3^)	m_eff_ (emu)
**ZnO**	0.03247	0.00734	1.06799 × 10^14^	3.0403 × 10^−16^
**ZnFe1**	0.04604	0.00678	1.6394 × 10^14^	2.8083 × 10^−16^
**ZnFe3**	0.06289	0.00674	2.25269 × 10^14^	2.7918 × 10^−16^
**ZnFe5**	0.0566	0.00674	2.02738 × 10^14^	2.7918 × 10^−16^

**Table 2:** Values of specific capacitances reported by various research groups.

S. No.	Material	Specific Capacitance (F/g)	Reference
1	Nd-doped ZnO	154	Sahu et al. [6]
2	ZnO Tetrapod	160.4	Luo et al. [22]
3	Graphene/ZnO	146	Lu et al. [28]
4	ZnO Nanowire	191.5	He et al. [31]
5	α-Fe_2_O_3_ Nanoparticles	603	Duan et al. [39]
6	MnO	545	Guo et al. [40]
7	NiCo_2_O_4_ Nanosheets	1730	Yang et al. [41]
8	ZnCl_2_ Porous Carbon Fibers	105	Duan et al. [42]
9	N-Doped Honeycomb-like Porous Carbon	275	Wang et al. [43]

## Data Availability

Available on request.
